# Earning Trust Before the Crisis: Health Literacy and Communication Readiness in Healthcare Systems

**DOI:** 10.3390/healthcare14172869

**Published:** 2026-09-07

**Authors:** Lorraine S. Evangelista

**Affiliations:** Sue & Bill Gross School of Nursing, University of California, Irvine, CA 92697, USA; l.evangelista@uci.edu

**Keywords:** public health preparedness, risk communication, organizational health literacy, community engagement, public trust, healthcare resilience

## Abstract

Risk communication and community engagement are fundamental to healthcare preparedness, yet many systems still focus solely on communication during a public health emergency. At this point, trust may be fragile, misinformation may be circulating, and healthcare professionals may be asked to explain changing guidance without sufficient preparation or organizational support. This Perspective starts from the premise that communication and community engagement should begin before a crisis. We argue that messaging alone is insufficient to earn trust; healthcare systems must be transparent, accessible, consistent, responsive, and equitable. Health literacy should not be viewed solely as an individual responsibility. We suggest five strategies to build communication readiness: (1) Listen to communities. (2) Prepare healthcare professionals. (3) Partner with trusted organizations. (4) Adapt communications to different populations. (5) Evaluate understanding of messages and support for protective action. Healthcare organizations must improve the access, understandability, and usability of information and services for populations with language barriers, disabilities, limited access to digital technologies, cultural differences, and inequitable access to care. These approaches need to be embedded in routine healthcare operations, not only during emergencies. Embedding communication readiness, organizational health literacy, and organizational trustworthiness into routine preparedness may provide a foundation for improving public understanding, equitable access to information, and healthcare resilience; however, these relationships require further study.

## 1. Introduction

Public health emergencies test more than a healthcare system’s supplies, staffing, surveillance, and clinical capacity. They also test whether the system can clearly explain a threat, respond to uncertainty, address public concerns, and help people take appropriate action. Dettori argues that risk communication, community engagement, and public trust should be treated as central parts of healthcare preparedness rather than added after an emergency has begun [1]. He also emphasizes that information alone is insufficient when people distrust the source or when communication overlooks fear, stigma, culture, and lived experience [1].

We support this position but believe it raises an important practical question: What must healthcare systems build before a crisis so that trusted and useful communication is already possible when an emergency occurs? Recognizing the importance of communication is not the same as being ready to communicate. Healthcare organizations need trained professionals, clear roles, consistent information sources, community feedback systems, trusted partnerships, and plans for responding to misinformation. The World Health Organization (WHO) describes risk communication and community engagement as work that should occur before, during, and after public health emergencies. Its recent competency framework also calls for a prepared workforce with the knowledge and skills needed to communicate and engage with communities effectively [2].

Health literacy provides an important organizational lens for this work. During an emergency, people may be asked to understand unfamiliar risks, evaluate changing information, decide which sources to trust, and follow recommendations while experiencing fear or uncertainty. Accurate information has limited value when people cannot access, understand, or use it. Health literacy should therefore not be viewed only as an individual ability. Healthcare systems also have a responsibility to ensure that their information and services are clear, accessible, and usable. Communication, navigation, organizational culture, policies and practices, community engagement, and workforce development are important elements of organizational health literacy [3].

Trust is more than the result of a carefully crafted message. It is created by honest communication, transparent explanations of uncertainty, equitable access to services, listening to community needs, and delivering on promises. Trust cannot be created out of thin air once a crisis is underway. It must be built over time through the routine actions and relationships of healthcare organizations.

Several established frameworks already address important parts of communication preparedness. The Centers for Disease Control and Prevention’s (CDC) Crisis and Emergency Risk Communication framework provides principles and practices for communicating during emergencies [4], while Public Health Emergency Preparedness Capability 4 addresses the development, coordination, and dissemination of emergency public information and opportunities for public interaction [5,6]. The WHO’s Risk Communication and Community Engagement guidance emphasizes community-centered communication and engagement throughout the emergency cycle [2]. Health literacy frameworks, including the Health Literate Care Model [7] and the attributes of health-literate healthcare organizations [8,9] emphasize organizational responsibility for making information and services understandable and usable. Together, these frameworks provide an important foundation for communication preparedness, but they differ in their primary purpose, setting, and unit of action. Table 1 compares these established frameworks with the five-action communication-readiness approach and highlights their differing purposes, settings, and units of action.

As a Perspective, this article does not use a formal systematic or scoping-review methodology. Rather, it draws on established risk-communication and emergency-preparedness guidance, organizational health-literacy frameworks, and selected recent empirical literature to provide a practice-oriented synthesis for healthcare delivery organizations.

This Perspective does not propose a new theoretical framework. Rather, it integrates these established principles and applies them specifically to healthcare organizations, including hospitals, clinics, and integrated health systems. These organizations differ from public health authorities in that they often interpret, operationalize, and deliver public health guidance rather than issue it. For this Perspective, communication readiness refers to the organizational capacity to receive, interpret, adapt, deliver, and evaluate timely health information through trusted relationships and accessible communication systems before and during a public health emergency. We therefore propose five practical actions for healthcare organizations: listen to communities, prepare healthcare professionals, partner with trusted organizations and community leaders, adapt communication to the needs of different populations, and evaluate whether communication is understood and supports protective action. Together, these actions translate existing guidance into a practical approach to routine organizational communication readiness before the next public health emergency.

## 2. Trust Cannot Be Communicated into Existence

Trust is often described as a goal of risk communication. This can create the impression that trust can be gained mainly through clearer or more convincing messages. Trust is also built on the actions of healthcare systems, though clear communication is important. People notice whether leaders communicate promptly, explain what is known and unknown, correct mistakes, provide fair access to services, and respond to community concerns. When a system’s actions do not match its messages, even well-designed communication may have little effect.

Trust can refer to confidence in an individual healthcare professional, a healthcare organization, a public health agency, or science more broadly. These forms of trust are related but are not the same. In this Perspective, our focus is primarily on healthcare organizations and their trustworthiness. Trustworthiness reflects whether an organization demonstrates competence, honesty, fairness, transparency, and responsiveness through its actions. From this perspective, the goal is not simply to make people more trusting. It is to help healthcare organizations act in ways that deserve trust [10,11].

Dettori similarly argues that risk communication is less effective when institutions lack credibility or fail to consider the broader context in which people receive and interpret health guidance [1]. Trust should therefore not be treated simply as a communication outcome. Healthcare organizations should therefore focus first on demonstrating trustworthiness through transparent, consistent, accessible, responsive, and fair practices. Trust may be weakened when recommendations change without explanation, when organizations provide conflicting guidance, or when people are asked to take unrealistic actions because needed services or resources are unavailable.

Healthcare systems must also avoid assuming that distrust is solely the product of misinformation or a lack of understanding. Sometimes these concerns are rooted in real experiences of discrimination, lack of access to care, lack of language support, or exclusion from decisions about care. In this situation, the lack of trust is unlikely to be resolved by providing more information. Healthcare organizations should begin by listening to people’s understanding of the threat, the barriers they face, and how their past experiences influence their responses. The WHO encourages community engagement and feedback channels to grasp concerns and collaboratively develop solutions [12].

Trust is built over time, in ongoing relationships, not in an emergency. Healthcare systems that develop relationships with community organizations, engage communities in planning, listen to feedback, and communicate openly in normal times will be more likely to have trusted pathways in place during a crisis. A recent systematic review found that trust, collaboration, tailored communication, inclusion, equity, and community feedback are closely connected with community resilience during health emergencies [13]. The goal should not be to convince communities to accept decisions that have already been made. The goal should be to create conditions in which healthcare systems and communities can understand risks, discuss concerns, and develop workable responses together. Trust cannot simply be created out of thin air after a crisis begins. It has to be earned by the daily choices, behaviors, and relationships of healthcare organizations.

## 3. Health Literacy Is Also an Organizational Responsibility

During a public health emergency, people may be asked to understand an unfamiliar threat, follow changing recommendations, compare conflicting information, and make decisions while worried about themselves or their families. Even people who usually manage health information well may find this difficult. When communication fails, however, the problem is often described as limited health literacy, lack of knowledge, or unwillingness to follow guidance. While this puts a heavy burden on the individual, it ignores the demands imposed by the health care system itself.

Organizational health literacy provides an alternative perspective. It asks not only whether people can understand health information, but also whether healthcare organizations make information and services easy to find, understand, and use [3,14]. This is important because health literacy is not only about individual skills. It is also about the clarity of messages, the organization of services, the availability of supports for language and disability, and the realism of the proposed action.

Information quality during an emergency involves more than scientific accuracy. Information must also be timely, consistent, transparent about uncertainty, accessible to the intended audience, and usable in the decisions people are being asked to make. People need to know what the message means for them, and what they should do next. A technically correct message can also fail if it is presented in an unfamiliar language, contains too much information, is communicated through inaccessible channels, or fails to explain why recommendations have changed. Similarly, telling people to seek testing, vaccination, treatment, or emergency care has limited value when those services are difficult to locate, afford, reach, or navigate.

The same message will not work equally well for every population. Some people may need information in their language, interpretive services, visual or audio formats, simplified instructions, or communication through a trusted community source. Others may lack internet access, have difficulty using digital platforms, have concerns stemming from prior healthcare experiences, or have responsibilities that make the recommended action difficult. Adapting communication to these realities does not weaken the message. It makes the message more usable and more equitable.

Organizational health literacy therefore belongs within healthcare preparedness. It should be reflected in communication plans, workforce training, emergency policies, digital platforms, service navigation, and community partnerships before a crisis occurs [3,8,9]. The question should not be, “Why did people fail to understand or follow the message?” A more useful question is, “What could the healthcare system have done to make the information and the recommended action clearer, more accessible, and more realistic?” Asking that question shifts preparedness away from blaming individuals and toward building systems that help people act.

## 4. Recognizing the Limits of Communication Readiness

Communication readiness can help healthcare organizations prepare for public health threats, but it cannot eliminate all distrust or guarantee that people will follow recommendations. Trust in healthcare and public health institutions may already be influenced by political beliefs, prior experiences with healthcare or government, social networks, and attitudes toward science and authority. Sometimes, people may form a judgment about whether they trust the messenger even before they hear the message. Open communication, community engagement, and transparency are still important, but may not be enough when distrust is deeply rooted [15,16].

There are also practical trade-offs. Maintaining community partnerships, training healthcare professionals, adapting materials, and evaluating communication all require time and resources that must compete with other preparedness priorities. Sustained investment may also be difficult to sustain because preparedness funding often increases after a crisis and then declines as public attention fades [17,18]. Healthcare organizations therefore need to decide which communication-readiness activities should be maintained routinely and which can be expanded when a threat emerges.

Community engagement can also create problems if it is not done well. Repeatedly asking communities for input without showing how that input was used can lead to frustration and partner fatigue. Healthcare organizations may also risk using up the trust that community partners have built if they rely on them too heavily without following through on commitments [19]. Trusted messengers also need timely and accurate information so they do not unintentionally share incomplete or incorrect guidance. Partnership should therefore involve ongoing communication, shared expectations, and clear pathways for correcting information when needed [20].

Finally, public health emergencies often require rapid decisions. Consultation and adaptation take time, and there may be situations in which healthcare organizations must act before broad community input can be obtained. Communication readiness should not be understood as a requirement to delay urgent action. Rather, the goal is to establish relationships, feedback channels, and communication processes before a crisis so that organizations can act quickly while still listening, explaining uncertainty, and adjusting their response as new information becomes available.

These limitations reinforce an important point: the five actions proposed in this Perspective should not be viewed as a guarantee of trust or cooperation. They are organizational practices intended to strengthen trustworthiness and communication capacity under conditions that may remain uncertain, politically contested, and resource-constrained.

## 5. From Principle to Practice: Five Actions for Communication Readiness

Recognizing that trust, health literacy, and communication are important is only the first step. Healthcare systems also need practical ways to build these capacities into everyday operations. We propose five connected actions: listen, prepare, partner, adapt, and evaluate. Together, these actions can help healthcare organizations strengthen communication readiness before a crisis and respond more effectively when public health threats emerge. Communication readiness can be assessed through observable structures, processes, and outcomes associated with each of these actions. Table 2 describes indicators that healthcare organizations may consider when assessing their readiness.

**Figure 1:**
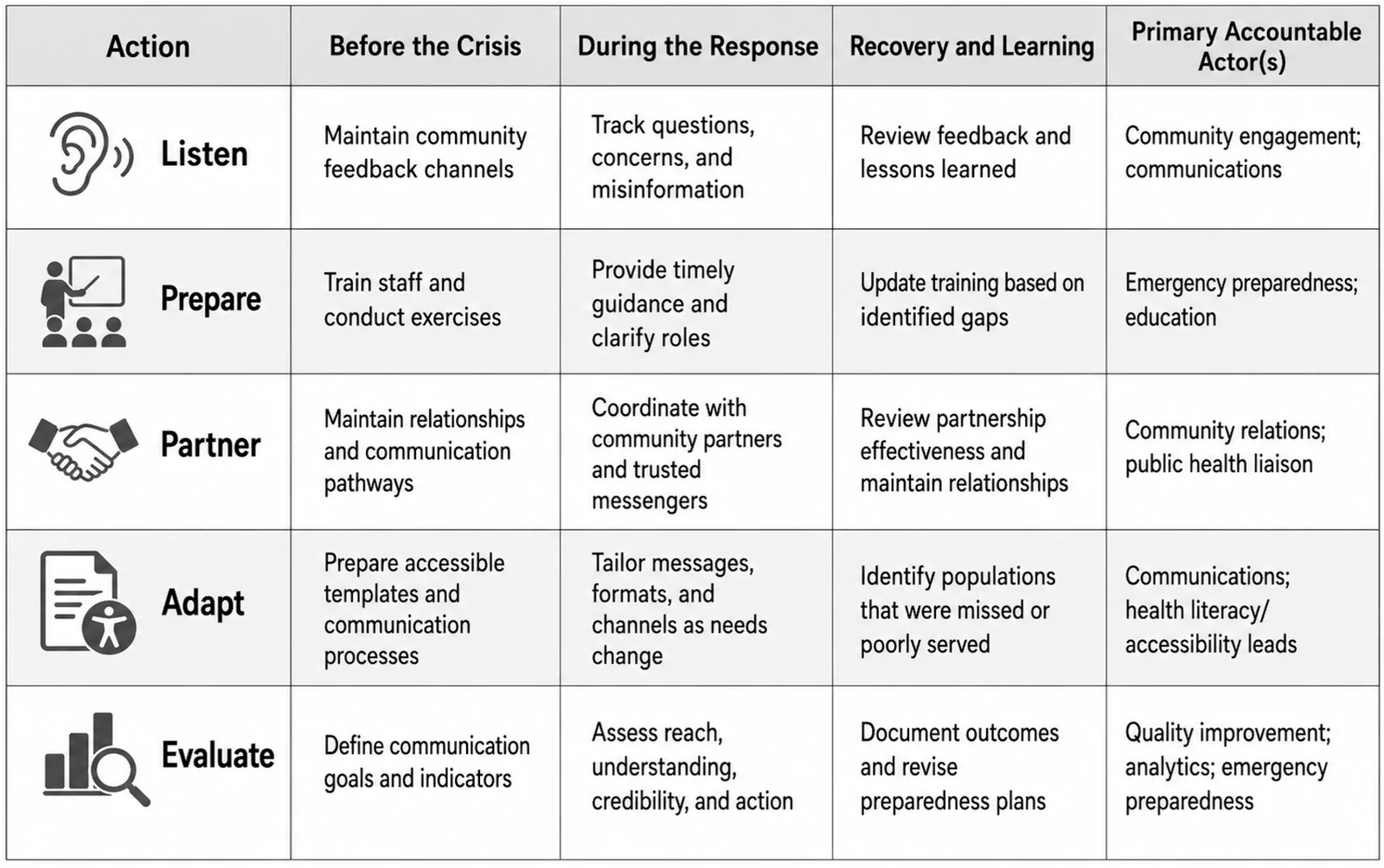
Communication readiness across the public health emergency cycle. The five actions—listen, prepare, partner, adapt, and evaluate—should operate before, during, and after a public health emergency rather than being activated only during crisis response. Organizational responsibilities may vary by setting. Illustrative indicators for each action are presented in Table 2.

Figure 1 shows how the five actions can be incorporated before, during, and after a public health emergency and identifies the organizational functions most likely to share responsibility for each action. Illustrative indicators for assessing communication readiness are presented in Table 2.

### 5.1. Listen

Healthcare systems often begin communicating by asking, “What do people need to know?” An equally important question is, “What do we need to understand about the people and communities we serve?” Listening should come before message development and continue throughout an emergency. Without it, healthcare organizations may provide accurate information that fails to address the questions, fears, or practical problems that affect people’s decisions.

Listening can help healthcare systems identify concerns before they become larger barriers. Communities may be worried about the safety of a recommended action, confused by changing guidance, uncertain about where to access services, or unable to follow recommendations due to cost, transportation, work, caregiving responsibilities, language, or limited digital access. These concerns should not automatically be dismissed as resistance or misinformation. They may point to weaknesses in the message, the service, or the system delivering it.

Community listening should use more than one source. Feedback may come from patients and families, healthcare professionals, community health workers, faith and cultural organizations, local leaders, call centers, surveys, community meetings, traditional media, and digital platforms. The WHO describes social listening as the careful review of conversations, questions, concerns, beliefs, and areas of uncertainty that may influence how people respond to a health threat [21]. Social listening can add useful information, but it must be conducted ethically and should not replace direct relationships with communities.

Listening also requires a clear way to respond. Collecting feedback has little value unless concerns reach decision-makers and lead to changes in communication or services. Healthcare organizations should decide in advance who will review community feedback, how urgent concerns will be shared, and how messages or services will be adjusted. They should also report back to communities so people can see that their input was heard and explain when a requested change cannot be made.

Most importantly, listening should not begin only when cooperation is needed. Healthcare systems should maintain feedback channels and community relationships during routine operations. Community feedback mechanisms should be part of preparedness and risk communication plans, according to the WHO [12]. Regular listening helps organizations understand the communities they serve, identify shifts in trust and information needs, and respond faster when a new threat emerges. It changes communication from something healthcare systems deliver to communities to something they develop with communities.

### 5.2. Prepare

Healthcare professionals are often expected to become trusted communicators as soon as an emergency begins. They may need to explain a new threat, address fear, correct misinformation, and discuss recommendations that are still evolving. However, many may be unprepared for these responsibilities. Knowing how to provide clinical care does not automatically prepare someone to communicate risk or uncertainty during a public health emergency.

Preparation should begin by clarifying roles. Healthcare professionals need to know where official information will come from, who is responsible for updating guidance, how questions should be reported, and where patients and families can be referred for additional help. Without clear roles and dependable sources, different professionals may provide different answers. This can lead to confusion and place people in the difficult position of having to explain decisions they do not fully understand.

Training has to be hands-on, not just about policies or written materials. Healthcare workers need to learn to speak in plain language, talk about what is known and what is not known, respond to tough questions, spot misinformation, and adapt communications for different populations. They also need to validate concerns, address them, and keep their promises. The WHO’s competency framework identifies the skills and behaviors needed for effective risk communication and community engagement before, during, and after emergencies [2].

Short simulations, case discussions, and communication exercises also enable teams to identify unclear roles, conflicting messages, and missing resources before a real emergency. These exercises should include situations in which evidence changes, services are limited, or a community does not immediately accept the recommended action. The goal is not to memorize one approved script. It is to learn how to communicate honestly and consistently when the situation is uncertain.

Healthcare organizations also need to keep their workforce informed and engaged. Professionals who interact directly with patients and communities often hear questions and concerns before organizational leaders do. They should have a simple way to share what they are hearing and receive timely answers. Recent WHO guidance emphasizes that health and care workers should be actively involved in preparedness and response because they are important partners in risk communication, community engagement, and the management of misinformation [22].

Finally, preparation requires organizational commitment. Training is unlikely to be effective when healthcare professionals lack time to participate, expectations differ across departments, or communication is treated as an individual skill rather than a shared responsibility. Recent research has noted wide variation in healthcare emergency-preparedness training and called for more consistent training standards [23]. Healthcare organizations should therefore assess communication readiness regularly, incorporate it into emergency exercises, and provide ongoing learning rather than waiting until a crisis is already underway.

### 5.3. Partner

Healthcare systems cannot build trust through internal planning alone. They need relationships with the people and organizations that already understand and serve their communities. These may include public health agencies, community health workers, faith and cultural organizations, schools, social service agencies, advocacy groups, local leaders, and organizations serving populations at greater risk during emergencies.

These relationships should be developed before a crisis begins. Healthcare systems often reach out to community organizations solely when they need assistance in sharing urgent messages or encouraging adherence to recommendations. This approach depends heavily on community partners as conduits for disseminating information. A true partnership begins earlier and provides community members a meaningful role in identifying concerns, setting priorities, developing messages, planning services, and determining how to allocate resources.

Real-world responses illustrate how these principles can work in practice. During the 2022–2023 UK mpox outbreak, community organizations and local sexual health clinics worked with affected populations to counter stigma and support health-seeking behavior; this experience highlighted the value of timely and inclusive collaboration among communities, healthcare providers, and public health authorities [19]. Similarly, the CDC Prevention Research Center Vaccine Confidence Network engaged trusted messengers with established community relationships across multiple U.S. sites and supported them with evidence-based information and training to inform COVID-19 vaccine activities [20]. These examples show how trusted relationships and usable information can become part of preparedness rather than being improvised after a crisis begins.

Community partners know things healthcare organizations do not. They often understand which messengers are trusted, which words may cause confusion or concern, where people receive information, and what barriers may prevent them from following public health guidance. They can also help healthcare systems recognize when a proposed response may not be realistic for the people expected to implement it. Their contribution should therefore begin during planning, not after major decisions have already been made.

Partnership also requires shared responsibility. Healthcare organizations should clarify roles, communication pathways, and decision-making processes before an emergency. Community partners may need training, up-to-date information, funding, technology, translation support, or other resources to participate fully. Asking organizations to contribute their time, relationships, and credibility without providing support can weaken rather than strengthen the partnership. The WHO’s guidelines for civil society engagement call for inclusive, community-centered decision-making and shared accountability throughout emergency prevention, preparedness, response, recovery, and resilience [24].

Health systems should also never rely on a single person or entity to speak for an entire community. Communities are diverse, and health experiences differ by age, language, culture, disability, place, income, immigration history, and access to care. By working with many, health systems can hear more concerns and decrease the chances that some voices will be drowned out.

Solid partnerships are built on regular contact, shared work, and follow-through in ordinary times. Healthcare organizations should remain engaged well after the immediate threat has passed, report back on how they used community input, and continue to address health and access-to-care issues. Existing partnerships enable healthcare systems to sidestep the scramble to find trusted messengers when a crisis hits. The relationships, communication channels, and shared understanding are already in place.

### 5.4. Adapt

Healthcare systems often try to create one clear message for everyone. Consistency is important, but the same message may not be understood or applied in the same way across all populations. Communication readiness requires healthcare organizations to adapt information without altering the core evidence or recommendations.

Adaptation involves more than translating written materials into another language. Healthcare systems may also need to change the wording, format, communication channel, messenger, timing, or level of detail. Some people may need plain-language instructions, pictures, captions, sign language interpretation, audio messages, large print, or help with using digital tools. Others may be more likely to receive and trust information shared through community health workers, faith organizations, local leaders, radio, text messages, or face-to-face conversations. Current public health preparedness guidance emphasizes that emergency communication materials should be accessible to people with disabilities, people with limited English proficiency, and others who face barriers to receiving information [25].

Healthcare organizations must also consider whether the recommended action is realistic. Advising people to seek testing, vaccination, treatment, or emergency care is not enough when services are far away, unaffordable, difficult to navigate, or unavailable outside working hours. Health literacy universal precautions provide a practical way to put this responsibility into practice. Rather than trying to identify who may have difficulty understanding health information, organizations assume that anyone may have difficulty and communicate clearly with everyone. This includes using plain language, limiting information to the most important points, providing appropriate language support, and confirming understanding through methods such as teach-back. Organizations can also assess their own health-literacy practices and identify areas for improvement rather than focusing only on an individual’s health-literacy skills [26]. Recent studies using measures of health-literate healthcare organization attributes also show that organizational health literacy can be assessed from the perspective of healthcare professionals [8]. Communication should clearly explain where people can go, what the service will cost, what documents may be needed, and what alternatives are available. When barriers cannot be removed, they should at least be acknowledged rather than ignored.

Adaptation should be guided by the people expected to use the information. Community members and healthcare professionals can help identify confusing language, inaccessible formats, cultural concerns, and recommendations that may be difficult to implement. Testing materials with the target audience before an emergency can uncover problems that the message developers may not see. Feedback should be continuous during a crisis so that communication can be modified to address changing needs and concerns.

Emergency planning, in particular, should focus on the excluded populations. These may include people with disabilities, older adults, migrants, rural communities, people with limited digital access, and those who do not speak the dominant language. The WHO recommends including disability needs within health-system governance, planning, and monitoring rather than treating accessibility as an added service [27]. The same principle applies more broadly: equity should be built into communication planning from the beginning, not added after gaps have already appeared.

Adapting communication does not mean giving different facts to different groups. It means making the same core guidance understandable, accessible, relevant, and possible to follow. A message is not truly effective because it was distributed widely. It is effective when the people who need it can receive, understand, and use it to make informed decisions.

### 5.5. Evaluate

In healthcare, we often evaluate communication by counting the number of messages sent, materials disseminated, website hits, or social media views. These metrics can tell you whether information was shared, but not whether people understood it, trusted it, or were able to act on it. Communication should be evaluated by what it helps people do, not only by how widely it is distributed.

Evaluation should start before a crisis by developing specific communication objectives. Healthcare organizations should decide what people need to know, what protective actions to take, which populations to reach, and how to identify and address community concerns. The Centers for Disease Control and Prevention recommends establishing outcomes for emergency risk communication, assessing whether those outcomes are achieved, and measuring whether information is shared equitably [25].

Healthcare systems should examine several questions. Did the information reach the people most affected by the emergency? Could they understand what the message meant and what they were expected to do? Did they view the source as credible? Did they receive services and/or referrals? Were some groups served later or less successfully than others? These questions move the evaluation beyond message dissemination to understanding whether the communication was useful and fair.

Community feedback should be part of the evaluation. Surveys and digital measures can be informative, but they may not tell us why a message was accepted, questioned, or ignored. Patients, families, healthcare professionals, and community partners can help identify ambiguous language, contradictory guidance, unmet needs, and barriers that communication data alone may not reveal; their feedback can also tell us whether changes made during the response improved understanding or access.

Evaluation should be ongoing during an emergency, not at the end. As information is gathered, services are delivered, and new issues are identified, the need for communication can change rapidly. Healthcare organizations should leverage lessons learned to adapt messaging, improve services, and identify gaps where there is an opportunity to influence outcomes. After the emergency, lessons learned should be documented and incorporated into future training, plans, and exercises. Centers for Disease Control and Prevention guidance recommends testing communication strategies during preparedness exercises and documenting lessons learned from their performance [25].

Not every communication problem can be prevented, and evaluation should not be used mainly to assign blame. Its purpose is to support learning. Healthcare systems should ask not only whether the public followed the guidance, but also whether the system provided information and services that people could understand, trust, access, and use. Evaluation should lead back to listening and continued improvement rather than mark the end of the process. As shown in Figure 1, healthcare systems must listen, prepare, partner, adapt, and evaluate to build communication readiness before the next crisis.

## 6. Future Directions: Testing Communication Readiness

The five actions proposed in this Perspective are grounded in existing guidance and established organizational practices. However, an important question remains: does greater communication readiness before a crisis actually improve outcomes during an emergency? This relationship should not be assumed. It should be tested. Future studies could examine whether healthcare organizations with stronger pre-crisis communication readiness are better able to reach communities, communicate changing guidance, support protective action, and reduce differences in access and understanding across population groups.

Several research methods may be useful. Prospective studies could assess communication readiness during routine operations and then examine organizational and community outcomes during an emergency. Natural experiments could compare healthcare organizations facing the same regional or public health threat but with different levels of readiness. Implementation studies could assess the feasibility of the five actions for sustainability during routine operations, and simulation studies could examine communication processes prior to an actual emergency.

Healthcare organizations can also begin collecting measures that would make this research possible. These could include communication reach and comprehension, perceived organizational trustworthiness, time from the emergence of a concern to organizational response, accessibility of information, uptake of recommended actions, and differences in these outcomes across population groups. The indicators presented in Table 2 offer a starting point, but they are not a validated measure of communication readiness. Developing and testing such a measure is an important next step.

## 7. Conclusions: Earning Trust Before the Crisis

Healthcare systems cannot wait for a public health emergency to consider trust, health literacy, and communication readiness. By that time, misinformation may be proliferating, public confidence may be waning, and health professionals may be asked to explain changing guidance without the proper preparation or support.

Clearer messages do not replace trust. Trust is built on transparency, fairness, accessibility, consistency, and the willingness to listen. It also depends on whether people can understand the information they receive and realistically act on the recommendations. For this reason, health literacy and communication readiness should be treated as organizational responsibilities and as essential parts of preparedness.

Healthcare systems routinely assess staffing, supplies, surveillance, emergency operations, and continuity of services. They should give the same attention to whether their workforce is prepared to communicate, whether trusted community relationships are already in place, and whether information can reach people in understandable, culturally appropriate, and useful ways.

Earning trust before a crisis requires steady work during ordinary times. Healthcare organizations must listen, prepare, partner, adapt, and evaluate before the next emergency occurs. These efforts will not eliminate uncertainty or prevent all communication breakdowns. However, they can help create better conditions for cooperation, informed decision-making, equitable access to information, and community resilience in the face of public health threats.

## Figures and Tables

**Table 1:** Relationship of the Five-Action Communication-Readiness Approach to Established Frameworks and Guidance.

Existing Framework	Primary Focus/Unit of Action	Relationship to the Five Actions	What This Perspective Adds
CDC Crisis and Emergency Risk Communication (CERC)	Organizations and communicators responding to emergencies	Supports listening, preparation, audience adaptation, community engagement, and evaluation across crisis phases	Shifts attention toward what healthcare organizations should establish before a crisis as routine organizational capacity
CDC PHEP Capability 4: Emergency Public Information and Warning	State, local, tribal, and territorial public health preparedness systems	Includes public interaction, information exchange, message coordination, dissemination, and reaching populations with limited access to information	Translates public-health preparedness functions to hospitals, clinics, and integrated healthcare systems that often interpret and operationalize rather than issue public-health guidance
WHO Risk Communication and Community Engagement (RCCE)	Public-health programs, national authorities, RCCE practitioners, and community partners	Strongly aligns with listening, partnership, workforce preparation, adaptation, and community feedback	Integrates RCCE principles with organizational health literacy and healthcare-delivery responsibilities
Health Literate Care Model/Health-Literate Healthcare Organization principles	Healthcare organizations and clinical care systems	Supports workforce preparation, accessible communication, patient/community involvement, adaptation, and evaluation	Places organizational health literacy explicitly within public-health emergency communication readiness
Five-action communication-readiness approach proposed in this Perspective	Hospitals, clinics, and integrated healthcare organizations	Listen–Prepare–Partner–Adapt–Evaluate	Integrates emergency communication, community engagement, and organizational health literacy into a concise pre-crisis readiness approach for healthcare organizations

Note: CERC = Crisis and Emergency Risk Communication; PHEP = Public Health Emergency Preparedness; RCCE = Risk Communication and Community Engagement. The five-action communication-readiness approach is intended as an integrative, practice-oriented application of established principles rather than as a new theoretical framework.

**Table 2:** Illustrative Indicators of Communication Readiness in Healthcare Organizations.

Action	Examples of Organizational Readiness	Illustrative Indicators
Listen	Standing mechanisms for community input; feedback channels active during routine operations	Presence of a community advisory mechanism; number and type of active feedback channels; median time from concern to documented response; proportion of concerns communicated back to the community
Prepare	Workforce training; clear communication roles; access to current guidance; emergency communication exercises	Percentage of relevant healthcare professionals completing communication training; frequency of simulations; time required to distribute updated guidance; proportion of staff who can identify the official source of information
Partner	Sustained relationships with community organizations and trusted messengers	Number and diversity of active community partners; frequency of engagement outside emergencies; presence of formal agreements or defined communication pathways; proportion of partners involved in preparedness planning
Adapt	Processes for making information understandable, accessible, culturally appropriate, and usable	Percentage of priority materials available in needed languages and accessible formats; time required to adapt urgent messages; use of audience testing; documented changes made in response to community feedback
Evaluate	Defined communication outcomes and mechanisms for continuous learning	Reach across population groups; message comprehension; perceived credibility or trustworthiness; ability to identify the recommended action; uptake of protective actions; differences in outcomes across population groups

Note: The indicators shown are illustrative examples of structures, processes, and outcomes that may reflect communication readiness. They are not intended as a validated assessment instrument. Selection and interpretation of indicators should be adapted to the organization, population, and public health context.

## Data Availability

No new data were created or analyzed in this study. Data sharing does not apply to this article.
